# Spatio-spectral control of coherent nanophotonics

**DOI:** 10.1515/nanoph-2023-0651

**Published:** 2024-01-09

**Authors:** June Sang Lee, Nikolaos Farmakidis, Samarth Aggarwal, Bowei Dong, Wen Zhou, Wolfram H. P. Pernice, Harish Bhaskaran

**Affiliations:** Department of Materials, University of Oxford, Oxford, UK; Kirchhoff-Institute for Physics, Heidelberg University, Heidelberg, Germany

**Keywords:** active photonics, photonic spatio-spectral reconfiguration, phase-change materials

## Abstract

Fast modulation of optical signals that carry multidimensional information in the form of wavelength, phase or polarization has fueled an explosion of interest in integrated photonics. This interest however masks a significant challenge which is that independent modulation of multi-wavelength carrier signals in a single waveguide is not trivial. Such challenge is attributed to the longitudinal direction of guided-mode propagation, limiting the spatial separation and modulation of electric-field. Here, we overcome this using a single photonic element that utilizes active coherent (near) perfect absorption. We make use of standing wave patterns to exploit the spatial-degrees-of-freedom of in-plane modes and individually address elements according to their mode number. By combining the concept of coherent absorption in spatio-spectral domain with active phase-change nanoantennas, we engineer and test an integrated, reconfigurable and multi-spectral modulator operating within a single element. Our approach demonstrates for the first time, a non-volatile, wavelength-addressable element, providing a pathway for exploring the tunable capabilities in both spatial and spectral domains of coherent nanophotonics.

## 1 Introduction

**Figure 1:**
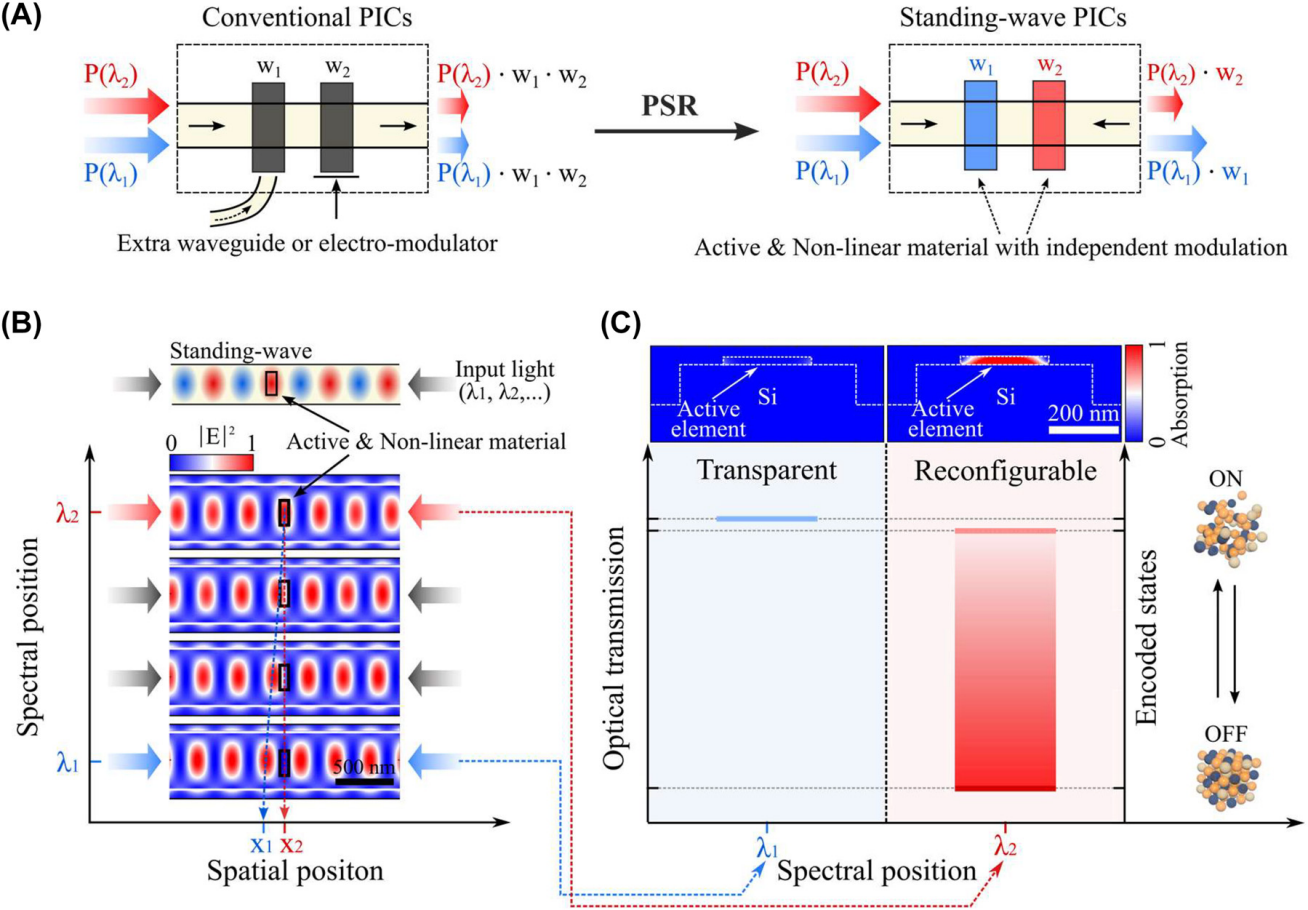
PSR in a single waveguide. (A) While conventional PICs (left) can carry the multiple optical signals at a single waveguide, the modulation (*w*_1_ and *w*_2_) of such signals is non-selective. However, standing-wave PICs (right) can bring the additional capability of selectively modulating wavelength-multiplexed signals without requiring extra waveguides or electro-modulators. This is enabled by photonic spatio-spectral reconfiguration (PSR) of active elements within the standing-wave cavities. (B) Schematic and electric-field distribution of an enlarged view for a single Si waveguide when wavelength-selective standing waves are formed due to interference of counter-propagating light (*λ*_1_ (1538 nm) – *λ*_2_ (1578 nm)). The active nanoantenna of 25 nm thickness, 100 nm width, and 250 nm length is placed on the waveguide. Spatial positions (*x*_1_ – *x*_2_) of standing waves are shifted at different spectral positions (*λ*_1_ – *λ*_2_), and such spatio-spectral modulation allows the light to pass through the nanoantenna undisturbed at one wavelength (*λ*_1_) but interacts with it at the other wavelength (*λ*_2_). Note that the field profile is an enlarged view from the full-field distribution in Supplementary Figure 1. Scale bar is 500 nm. (C) Wavelength-selectively reconfigurable optical transmission (i.e. absorption) is achieved at *λ*_2_ upon switching the nanoantenna between on (amorphous) and off (crystalline) states, while the absorption at *λ*_1_ is suppressed. Inset shows a cross-section of normalized absorption profile for a Si waveguide with the active nanoantenna, which exhibits wavelength-selective absorption. Scale bar is 200 nm.

Photonic integrated circuits (PICs) [1] can carry dense information by separating optical signals in space, wavelength and polarization. The enhanced bandwidth of photonics enables the efficient and compact realization of photonic cores [2], [3], neuromorphic network arrays [4], [5], [6], [7], broadband photodetection [8] and optical switches [9], [10]. Whilst photonic accelerators [11] have demonstrated a breadth of functions which can be achieved passively, reconfiguration remains a bottleneck. Active photonic circuits exploiting thermo-optic [12], electro-optic [13], [14] and electro-mechanical [15] mechanisms, have been implemented to reconfigure photonic circuits. However, due to the longitudinal propagation of guided modes in a waveguide, it continues to be challenging to select and separate multiple optical signals travelling within a photonic structure (see Figure 1A). Furthermore, the integration of active electronic circuitry thus far depends on the relatively weak optical response induced by thermo-optic and electro-optic mechanisms. This inevitably increases the overall device footprint and energy consumption. Importantly, such devices require long interaction lengths which annihilates wavelength or phase selectivity. There is thus a pressing need for techniques capable of producing a strong optical response within a sub-wavelength spatial domain to provide a pathway towards compact and selective modulation.

Standing waves are formed when counter-propagating light interferes in a cavity medium [16], where each standing wave is wavelength-decoupled. By modulating the spectral parameters (i.e. wavelength or phase), the spatial positions of the standing waves can be shifted so that the intensity contrast between two wavelengths is maximized at a specific position along the optical axis. Once the cavity is filled with an absorptive medium, wavelength-selective absorption, so-called coherent perfect absorption (CPA) [16], [17], [18], can be realized, enabling applications such as time-reversed lasing [19], [20] and optical switches [21], [22]. However, the properties of absorptive media have been static thus far, limiting the operation in passive mode. By employing active phase-change nanomaterials [23], [24] with standing-wave photonics, we demonstrate active CPA, and therefore spatio-spectral modulation of photonic devices. This concept enables independent modulation of wavelength-multiplexed optical signals travelling in a single waveguide (Figure 1A).

Here, we design on-chip photonic structures that can wavelength-selectively reconfigure the device states using two counter propagating waves through a weakly absorptive medium [16]. This operation relies on the formation of a standing wave when coherent light of nearly equal intensity is coupled to a waveguide or microring resonator from opposite directions. The mode number of the standing wave pattern defines the spatial distribution of nodes which can be addressed by wavelength or phase. By combining this effect with active phase-change nanoantennas [25], [26] opportunely positioned along the optical path, all-optical wavelength-selective modulation of the coherent absorption can be engineered, enabling so-called photonic spatio-spectral reconfiguration (PSR). We demonstrate this novel mechanism by demonstrating an intensity-reconfigurable, multi-wavelength all-optical modulator operating within a single element.

## 2 Results and discussion

The concept of on-chip PSR can be realized by exploiting the guided mode in a waveguide system with an active nanoantenna (Figure 1B and Supplementary Figure 1). We simulated a Si waveguide (finite-difference time-domain (FDTD), Lumerical) when light with two wavelengths (*λ*_1_ and *λ*_2_) propagates in opposite directions and the active phase-change nanoanntena (e.g., Ge_2_Sb_2_Te_5_ (GST) [23]) is placed on the waveguide. The field profile is obtained at a distance of 4 µm away from the centre of the waveguide along the propagation axis (Supplementary Figure 1). One can observe that the guided mode at each wavelength generates standing wave patterns, but with slightly different spatial periodicity. This causes an offset in positions (*x*_1_ and *x*_2_) of the standing wave maxima and minima (i.e. nodes) along the propagation axis of the waves. This arrangement forms the basis for a wavelength-selective system that the standing wave at *λ*_2_ is evanescently coupled with the nanoantenna while the other standing wave at *λ*_1_ passes through the nanoantenna unperturbed. The wavelength-selectivity can be uniquely combined with non-volatile tunability of our phase-change nanoantenna. As demonstrated in Figure 1C, the nanoantenna is fully reversible via non-linear threshold switching between on (amorphous; low *k*) and off (crystalline; high *k*) states [23], [27], [28], therefore we can reconfigure and store the optical states of the system selectively at the wavelength of interest (e.g., *λ*_2_).

**Figure 2:**
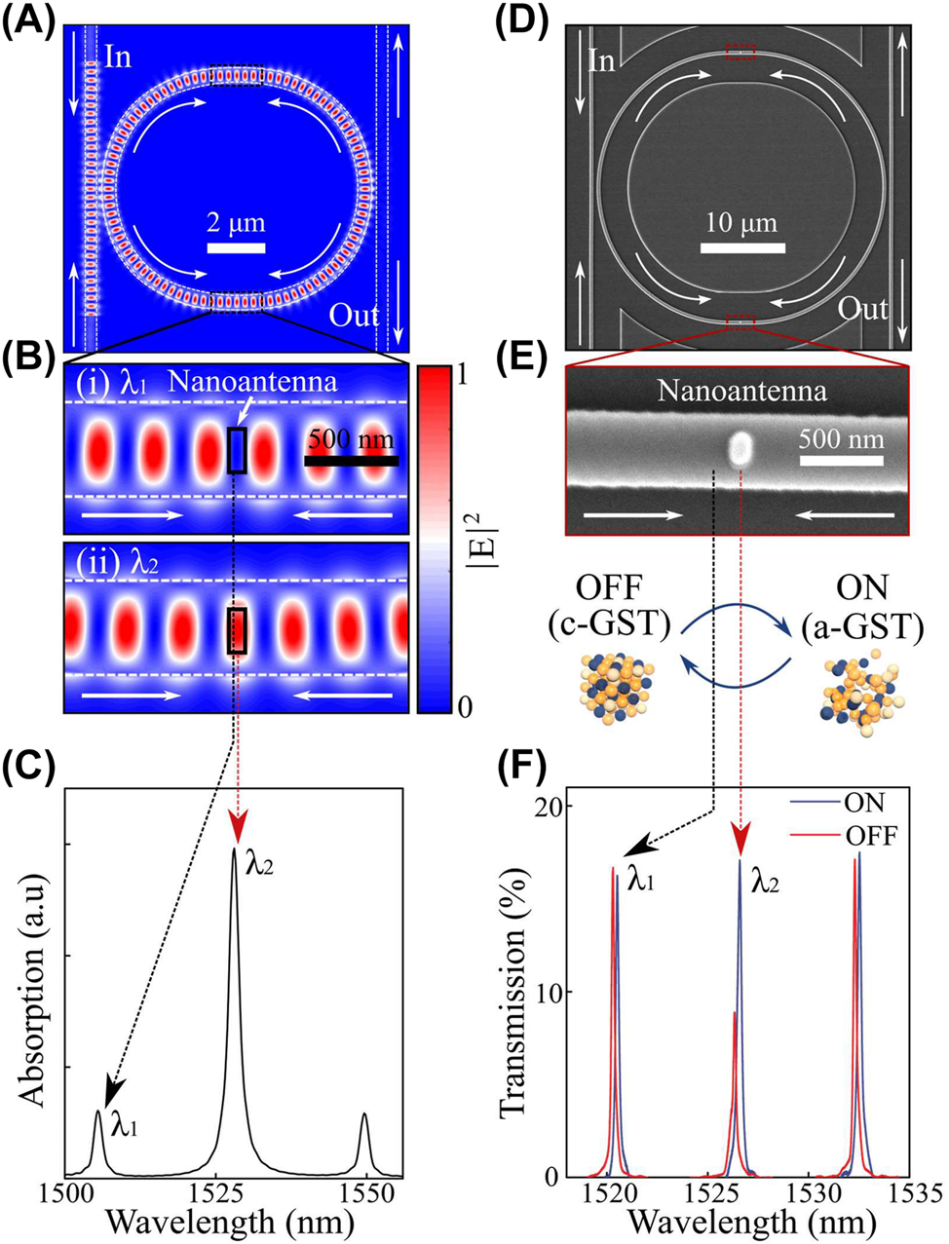
PSR in a microring resonator. (A and B) Electric-field distribution of a racetrack microring resonator when the counter-propagating waves from two input arms generate standing waves. Standing waves are out-of-phase depending on the incoming wavelength so that the active nanoantenna (i.e. GST) is invisible to the odd-mode (*top*, 1505 nm (*λ*_1_)), but evanescently coupled to the even-mode (*bottom*, 1528 nm (*λ*_2_)). Scale bars are 2 µm and 500 nm, respectively. (C) Absorption spectrum of the active nanoantenna that shows a selectively high absorption at the even-mode. (D and E) SEM scan of a racetrack microring resonator with active nanoantenna deposited on a waveguide. Scale bars are 10 µm and 500 nm, respectively. (F) Transmission spectra of a racetrack mirroring resonator when the nanoantenna is in (*blue*, ON) as-deposited amorphous and (*red*, OFF) crystalline phase after heating the device onto a hot-plate.

We now extend the concept of PSR from a waveguide to a resonant structure where the oscillation of the standing waves in the resonant cavity is employed to enhance the spectral and spatial resolution of the wavelength-selectivity. As shown in Figure 2A–C, we simulated the electric-field profiles at resonance modes of a microring resonator (variational-FDTD (varFDTD), Lumerical) when the incoming light is guided in opposite directions. The optical confinement of the standing waves is now enhanced by the multimode oscillations of whispering gallery modes (WGMs) in the microring resonator, where the periodic position (*P*) of standing wave is determined by the mode number of such resonances as shown below:

$$P=l.\lambda /2n_{\text{eff}}$$

where *λ* is the resonant wavelength, *l* is the position index along the microring resonator and *n*_eff_ is the effective refractive index of the waveguide. The mode numbers are mainly divided in two photonic modes of WGM resonances; odd- and even-modes which are manifested at resonant wavelengths in alternating order [17]. Because these modes exhibit out-of-phase standing waves at the midpoint of a microring resonator, the spatial overlap between the nanoantenna and one of the photonic modes determines the wavelength-selective coupling (Figure 2B). As shown in Figure 2C, we observe high absorption at the even-mode resonance (*λ*_2_), suggesting that the even-mode interacts with the nanoantenna while the odd-mode (*λ*_1_) propagates through the microring unaffected. This selectivity is an additional functionality that is unique to microring resonators with standing wave oscillations (Supplementary Figure 2).

We then proceed to demonstrate the concept of PSR experimentally. Figure 2D and E shows a scanning electron micrograph (SEM) of the fabricated devices (Detailed parameters are described in Methods and Supplementary Figure 3). The active GST/SiO_2_ nanoantenna of 100 nm width, 250 nm length and a combined thickness of 30 nm (25 nm GST at the bottom with 5 nm SiO_2_ on the top) is located at each arm of the microring to increase the interaction strength with standing waves. Two counter propagating waves in a microring-resonator generate standing waves and eventually outcouple to drop waveguides where the output transmission is monitored (see Methods and Supplementary Figure 4). The mode overlap between oscillating electric-field and nanoantennas leads propagating light to be attenuated by the insertion loss of nanoantennas in a wavelength-selective manner, where the insertion loss is tunable in our case upon the crystallization of GST. The optical transmission levels of selected modes are modulated by phase-change of the GST. Figure 2F shows the experimentally measured selective reduction of even-mode transmission (*λ*_2_) when the GST is switched from the as-deposited amorphous state to the lossy crystalline state after heating it onto a hot-plate at 200 °C for 5 min. The even-mode resonance (*λ*_2_) exhibits attenuation by 49.2 % while odd-mode (*λ*_1_) is unaffected ( ≤ 2.1 %), while the insertion loss is measured to be 3.2 ± 1.2 dB and trivial peak shifts are attributed to evaporation of polymer residue or moisture (described in Section S1 [29]−[33]). Also, the spectral selectivity of two modes (*λ*_1_, *λ*_2_) can be further optimized by fine-tuning the spatial positions of nanoantennas (described in Section S3). These results qualitatively agree with the calculated absorption spectrum in Figure 2C, although discrepancies in the spectral peak positions are observed because we use smaller sizes of the ring (*d* = 8 µm) for calculation. The wavelength-selective tunability of our system shows high resolution in both the spatial and spectral scale. The spatial resolution is ∼149 nm calculated by the separation of two standing waves (*λ*/4*n*_eff_), and the spectral resolution is ∼6.2 nm determined by free spectral range (FSR) of ring resonators.

**Figure 3:**
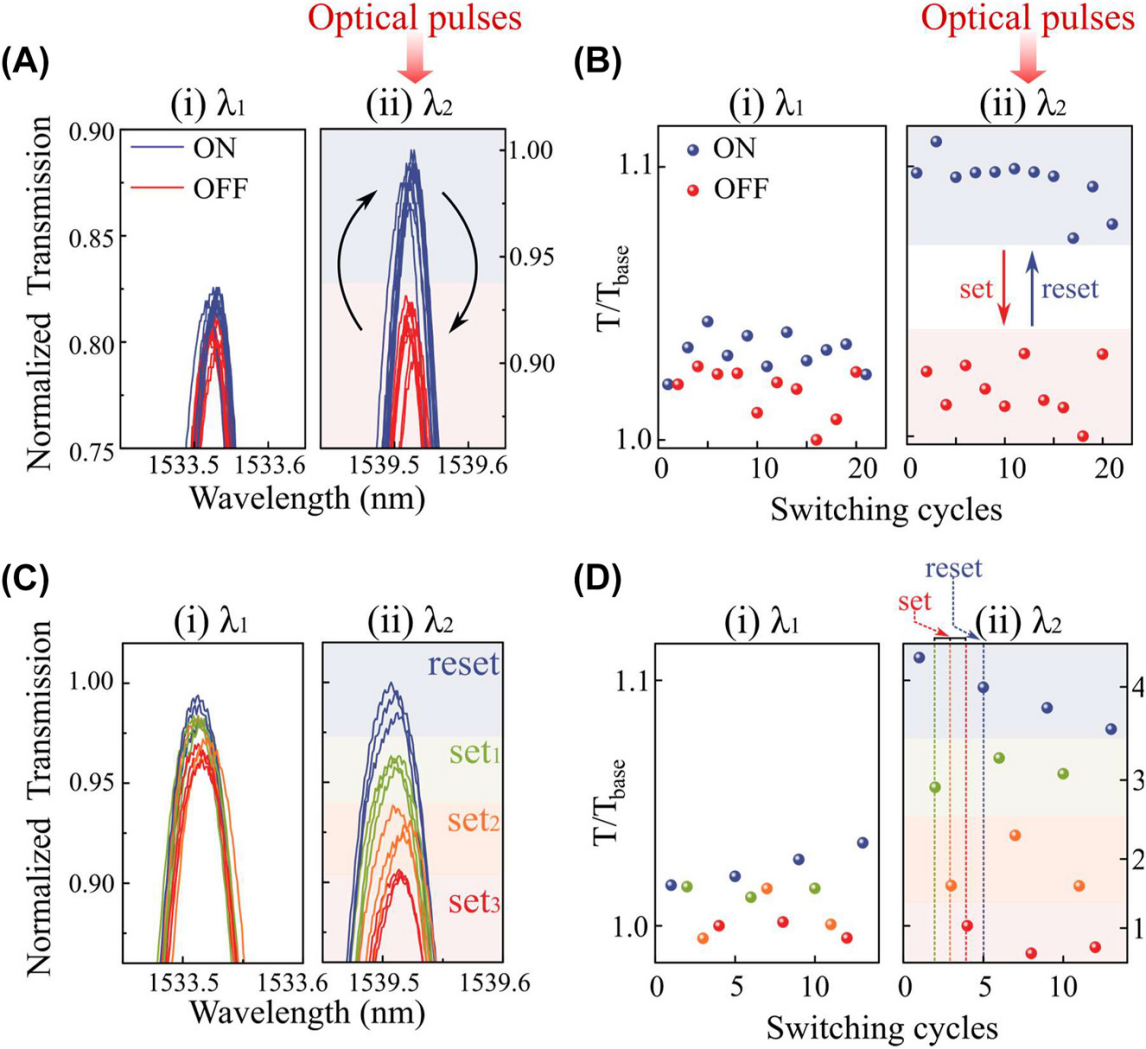
Wavelength-selectively reconfigurable system by optical pulses. (A) Enlarged transmission spectra of the PSR system with active nanoantennas (GST/Au) at the odd-mode (left, *λ*_1_) and the even mode (right, *λ*_2_), respectively. The set (220 pJ for 10 ns + 1.8 nJ for 300 ns with repetition of 50 times) and reset (220 pJ for 10 ns) optical pulses are sent to the device at the even-mode wavelength so that the reconfigurable transmission levels are only achieved in the even-mode wavelength. (B) Switching cyclability of wavelength-selective switching as in A. (C) Enlarged transmission spectra of multi-level wavelength-selective operations at the odd-mode (left, *λ*_1_) and the even-mode (right, *λ*_2_), respectively. The wavelength of optical pulses is at the even-mode and the number of such set pulses vary from 10 to 50. (D) Switching cyclability of wavelength-selective multi-level operations as in C.

We then investigate the capability to actively reconfigure the active nanoantennas using optical write pulses. Here, we exploit wavelength itself as a tunable factor to reconfigure the device states. Because the crystalline state of the GST is fully reversible [27], we can start a set of switching experiments from any states. For the experiments in Figure 3, amorphous phase is the initial state and we use GST/Au heterostructures on the partially-etched waveguide to enhance the absorptive coupling [17], [18], [34] (see Methods and Section S2). Since the nanoantennas are located at the spatial location of even-mode resonances (*λ*_2_), we illuminate the set (220 pJ for 10 ns followed by 1.8 nJ for 300 ns with repetition of 50 times at intervals of 10 µs) and the reset (220 pJ for 10 ns) pulses selectively at the even-mode wavelength (1533.5 nm) to achieve fully reversible switching. Since the exact spectral position of the microring resonances differs between devices due to our device fabrication tolerances (Section S1), a different pair of odd- and even-mode wavelengths is selected for each microring device. For set (crystallization) processes, double-step pulses are used to enhance the reliability of its reversible switching [35]. As a result, the reversible states are only observed in the even-mode wavelength (*λ*_2_, 1539.5 nm) while the odd-mode (*λ*_1_, 1533.5 nm) is unaffected (Figure 3A). The modulation contrast for the even-mode resonance remains around 10 %, whereas the one for the odd-mode is below 1.6 % (Figure 3B). When the laser pulses are sent to odd-mode resonances, the absence of a mode maximum induces negligible transmission contrast ( ≤ 2 %) in both modes (Supplementary Figure 6), thus confirming selective programmability. While 10 % of modulation contrast is close to the limit that nanoantennas in general can achieve (Section S2) [9], [28], [36], this can be further enhanced by tailoring the size, number, or material choice [37], [38] of nanoantennas (Supplementary Figure 7).

The optical transmission levels are also dependent on the fractional volume of the switched crystalline domains in the GST. Therefore, by controlling the energy of input pulses, we can achieve the variation of intermediate transmission levels [9], [35], [39], [40]. We vary the number of set pulses [3] from 10 to 50 to achieve multi-level crystallization (Figure 3C and D). Upon the excitation of even-mode pulses (1539.5 nm), our device exhibit 4 distinct multi-levels selectively at the even-mode wavelength, but not at the odd-mode wavelength (1533.5 nm). Such programmable multi-level operation is repeatable for multiple cycles. This is a significant improvement over a standard microring resonator which exhibits non-selective tunability at every wavelength.

**Figure 4:**
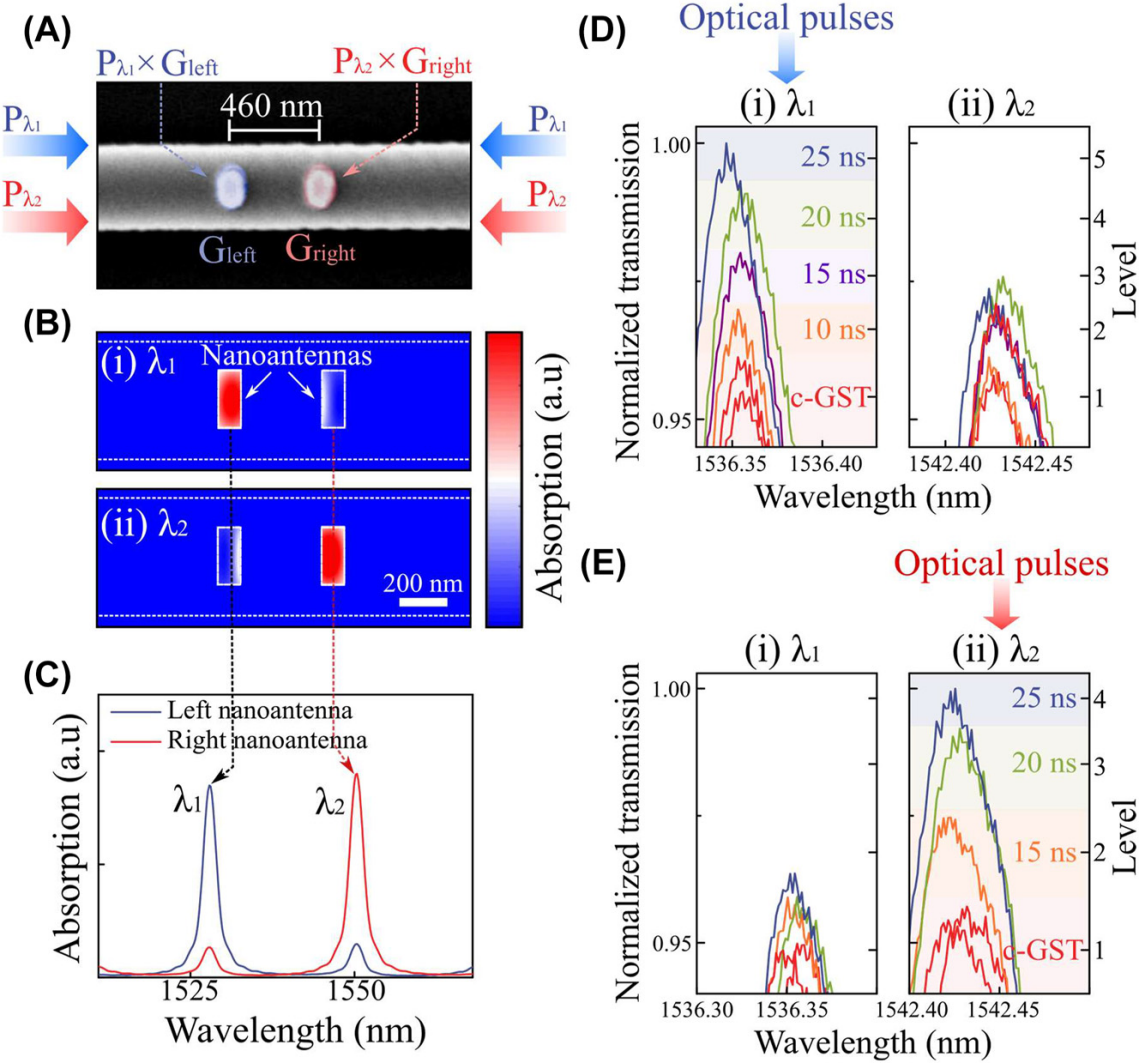
Reconfigurable multi-spectral filter in a single element. (A) Colorized SEM image of an enlarged view of the active nanoantennas (GST/Au) on a waveguide for PSR system. The separation between two nanoantennas is 460 nm. Wavelength-selective input signals (*P*_*λ*1_ and *P*_*λ*2_) are independently modulated by weighting coefficients of each nanoantenna (*G*_left_ and *G*_right_). (B) Absorption profile of the PSR system with double nanoantennas, where the odd-mode (*top*, 1528 nm (*λ*_1_)) couples with the left nanoantenna and the even-mode (*bottom*, 1550 nm (*λ*_2_)) couples with the right nanoantenna. (C) Absorption spectra at each nanoantenna showing the wavelength-selective absorption contrast. (D and E) Enlarged transmission spectra of intensity-tunable multi-spectral filter at the odd-mode (left, 1536 nm (*λ*_1_)) and the even-mode (right, 1542 nm (*λ*_2_)), respectively. Reset pulses of 220 pJ (10 ns) with varying the pulse duration from 10 to 25 ns are used to provide wavelength-selective multi-levels under (D) odd-mode and (E) even-mode illumination. The piecewise set pulse of (220 pJ for 10 ns + 1.8 nJ for 300 ns with repetition of 50 times) is used for initializing the transmission level.

We further extend the model of PSR to realize a reconfigurable and multi-spectral optical filter, with intensity independently controlled at each wavelength. As shown in Figure 4A, two sets of GST/Au nanoantennas are placed on a racetrack ring resonator with spacing of 460 nm (3*λ*/4*n*_eff_), such that they are positioned at each node of two photonic modes (i.e. odd- and even-modes). The mode-selective coupling induces wavelength-selective accessibility to individual nanoantennas, and therefore the intensities of wavelength-selective input (*P*_*λ*1_ and *P*_*λ*2_) are independently modulated by the wavelength-selective weighting coefficients (*G*_left_ and *G*_right_). Figure 4B and C shows that left and right nanoantennas exhibit selective absorptive coupling at odd-and even-mode resonances, respectively, resulting in the absorption contrast at each resonant wavelength. Such absorption contrast is used to set the threshold of selective switching for individual naonantennas. We experimentally verify this by showing the wavelength-selective multi-level operation (Figure 4D and E) and switching cyclability (Supplementary Figure 8) at each photonic mode. Upon odd-mode (*λ*_1_, 1530.3 nm) illumination, we note that the laser power is sufficient to selectively switch the left nanoantenna, but not sufficient to switch the right nanoantenna. Therefore, independent multi-level encoding can be demonstrated selectively (left nanoantenna, Figure 4D). In this case, we start in the crystalline state and control the reset pulse duration [35] from 10 ns to 25 ns to vary the fractional volume of the amorphized GST. By sending these pulses at the odd-mode resonance, five multi-levels are wavelength-selectively encoded without affecting the even-mode. This process is fully reversible as these multi-levels can be erased back to the initial state by high-power set pulse. The thermo-optic delay time [41] is neglected and this is a reasonable approximation, owing to the confined volume of GST nanoantennas on a thermally-conductive silicon waveguide [42]. Such wavelength-selectivity is also reproduced with reversed mode selections under even-mode illumination (*λ*_2_, 1536. 5 nm) as demonstrated in Figure 4E.

Thus, our reconfigurable multi-spectral filter provides independent accessibility to each nanoantenna with decoupled parameters. For example, our multi-spectral modulator can be utilized with a broadband light source such as supercontinuum laser [33] or optical frequency comb [43]. It is worth nothing that this approach is all-optical and non-volatile, providing a significant advantage over electro-optic or thermo-optical components (described in Section S3). Importantly, this functionality is achieved in a single on-chip photonic unit, instead of using multiple ring resonators [9] or free-space optical illumination [17]. Therefore, we demonstrate a compact building block of wavelength-selective photonic interconnects that can be exploited as reconfigurable optical filters [44], wavelength-division multiplexing/demultiplexing [45] or in-memory computing units [40]. Furthermore, such building block can achieve a dramatic enhancement of overall optical storage capacity as it gets upscaled, for instance by assembling multiple ring resonators in series (Supplementary Figure 15) or coupled to each other and tailoring their ring sizes (i.e. FSR). In this case, the precise control spatio-spectral overlap is essential which can be achieved by tuning the relative optical phases of two input light (Supplementary Figures 9–11). Phase shifters can be implemented outside a microring resonator by using thermo- or electro-optical modulators that enhance the robustness of system and provide extra degrees-of-freedom in phase domain.

Also, the concept of PSR is readily generalized to other photonic components such as plain waveguides or multimode interferometers (MMI) while the spectral selectivity is determined by the spatial location of active material along the propagation axis (Supplementary Figure 12). This spatio-spectral relation raises interesting possibilities for the exploitation of more than two wavelength-selective channels in a single microring resonator (Section S4.2). Although a tradeoff exists between device footprint and number of modulations, three independent modulations with the crosstalk of <−15 dB can feasibly be implemented (Supplementary Figure 13); this can be further improved by optimizing the geometrical or material properties of nanoantennas [38], [46], [47] (described in Section S2). However, as the number of modulation increases, the high-precision post-processing techniques [48] will be required to fine-tune the spectral resonance positions of microring resonators, compensating for its low fabrication tolerance. Because our PSR system allows for (de)multiplexing functionalities at highly confined scales, the overall modulation or multiplexing densities are found to be several orders of magnitude higher than those of other integrated multiplexing switches [49], [50], [51], [52] (described in Section S4.3). This wavelength-selective reconfigurability at sub-microns scale offers a promising avenue for programmable and compact photonic networks that can exploit large bandwidths or for sensing or imaging applications that require high-resolution spectral or spatial information.

## 3 Conclusions

In summary, we demonstrate a compact prototype of an integrated, wavelength-selective, reconfigurable device in which the intensity of light can be independently modulated at each wavelength channel of a single photonic element. Our approach exploits the concept of coherent perfect absorption with non-volatile tunability of phase-change material, thereby enabling the use of spatial degrees-of-freedom in integrated photonics and the realization of ultracompact reconfigurability, or PSR. Our device exhibits excellent spatial and spectral resolution by combining the WGM resonances in a microring resonator with active phase-change nanoantennas. The strong optical confinement of the electric-field within our nanoantennas allows for maintaining wavelength-addressability at highly confined spatial scales. Using this approach, we demonstrate a photonic, reconfigurable, multi-spectral filter at the single element level. Our results provide the groundwork for realizing functional photonic circuits having input wavelengths as variables and can serve as the building block of wavelength-multiplexed coherent nanophotonics, opening up a myriad of potential applications in neuromorphic networks, in-memory computing paradigms, optical communications, or high-resolution imaging applications.

## 4 Methods

### 4.1 Device fabrication

A first run of electron-beam lithography (EBL) was performed on a 220 nm Si on 3 µm oxide layer to fabricate waveguides, grating couplers, multimode-interferometers (MMIs) and microring resonators with using positive tone resist (CSAR62). After development, the patterned substrate was etched at a depth of 110 nm by reactive-ion etching (RIE, Oxford Instruments) with a gas mixture of CHF_3_/Ar/O_2_. A second EBL was carried out with double-layered PMMA resist to define the nanoantennas with widths of 100 nm and lengths of 250 nm on microring resonators. After development, the patterned substrate was subjected to a lift-off process followed by RIE at a depth of 30 nm and RF-sputtering (Nordiko) of GST/SiO_2_ of 25 and 5 nm thickness, respectively. For active switching experiments (Figures 3 and 4), additional Cr/Au layers of 3 and 20 nm thickness, respectively, were deposited in advance of a lift-off process to enhance the absorption of nanoantennas. We finely swept the positions of nanoantennas onto an array of microring resonators so that optimal spatio-spectral overlap can be obtained (Supplementary Figure 11). Also, in advance of active switching, the fabricated devices were heated on a hot-plate (200 °C) for 5 min to fully crystallize the GST.

### 4.2 Measurement setup

The measurement was carried out using the setup described in Supplementary Figure 4. A tunable laser (Santec, TSL-550) was used to optically probe the device at telecommunication range (1500–1600 nm), while the input and output grating couplers were optimized at 1550 nm wavelength. The coupled light was separated by MMIs at a ratio of 50:50 and recombined into a microring resonator. Optical transmission and spectral responses of drop waveguides were measured from output grating couplers using a 200-kHz low-noise photoreceiver (New Focus, Model 2011). For optical programming, the pump line was separated by optical splitter (90:10), which was modulated by an electro-optical modulator (EOM, Lucent Technologies) with an electrical pulse generator (AFG3102C, Tektronix). The generated optical pulses were amplified by an erbium-doped fiber amplifier (EDFA, HPP-PMFA-21-10 from Pritel) and monitored by a 125-MHz low-noise photoreceiver (New Focus, 1811) with an oscilloscope (Telktronix, TDS7404B). Rack optical switch (Gezhi) and circulator were used to separate the probe transmission from pump line for spectral measurement. Double-step pulses were used for a crystallization process which can produce more repeatable phase-change transition [35]. The number of pulses [3] was controlled for multi-level crystallization (Figure 3) and the pulse duration [35] was modulated for multi-level amorphization (Figure 4). Different microring devices were used for a single nanoantenna measurement in Figure 2F and 3. All of measurement with modulation of pulses was carried out by a custom-built program (Labview).

## Supplementary Material

Supplementary Material Details

Supplementary Material Details
