# Supplementary material for: Spatio-spectral control of coherent nanophotonics
Source: Nanophotonics. 2024 Jan 9;13(12):2117–25. doi: 10.1515/nanoph-2023-0651 (PMC11501196; doi:10.1515/nanoph-2023-0651)
Supplement: Supplementary file 2 — Supplementary Material Details [file j_nanoph-2023-0651_suppl_002.pdf]

Supplementary Information

for

"Spatio-spectral control of coherent nanophotonics"

June Sang Lee,[†,#] *Nikolaos Farmakidis,*[†,#] *Samarth Aggarwal,*[†] *Bowei Dong,*[†] *Wen Zhou,*[†] *Wolfram HP Pernice,*[‡] *and Harish Bhaskaran*[†,*]

[†]Department of Materials, University of Oxford, Oxford, UK

[‡]Kirchhoff-Institut für Physik, Universität Heidelberg, Heidelberg, Germany

[#]These authors contributed equally

*Corresponding author: Harish Bhaskaran: harish.bhaskaran@materials.ox.ac.uk

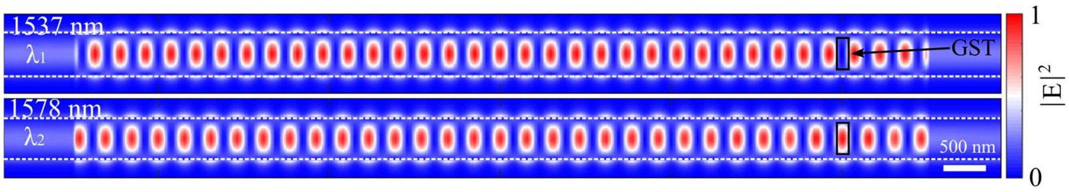

Supplementary Fig. 1. Simulated electric-field distribution at two wavelengths in a telecommunication range (1500-1600 nm). The light *passes through the* GST undisturbed at one wavelength (*top*, 1537 nm (λ_1)) *but interacts with the GST at the other wavelength (bottom*, 1578 nm (λ_2)*)*. Scale bar is 500 nm.

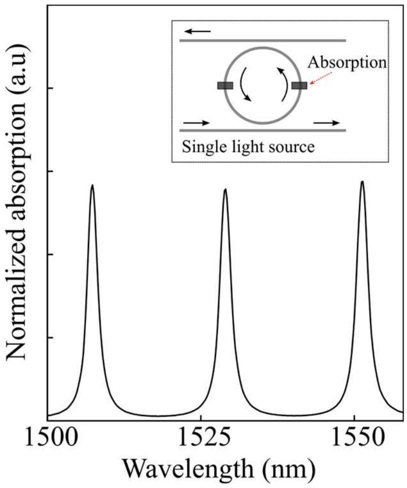

Supplementary Fig. 2. Absorption spectrum of GST nanoantenna when the light is guided from a single input source (i.e. conventional microring resonator).

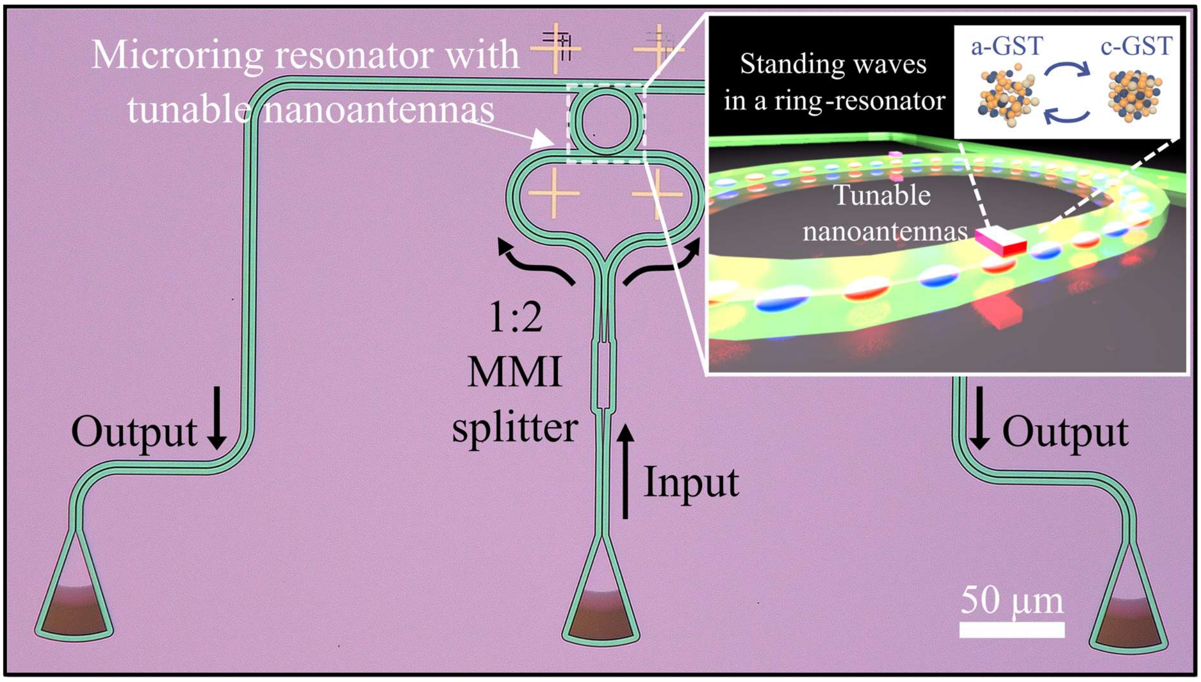

Supplementary Fig. 3. Optical microscope image of a microring PSR system. Input light at telecommunication range (1500-1600 nm) is guided into a 1:2 multimode-interference (MMI) splitter and combined into a microring resonator (diameter = 30 μm) from two opposite directions. Inset shows a microring-resonator with standing waves produced by two counter-propagating waves. Tunable nanoantennas (GST/SiO_2) are deposited on the microring resonator. Each layer of nanoantenna has 25 nm and 5 nm thickness, respectively.

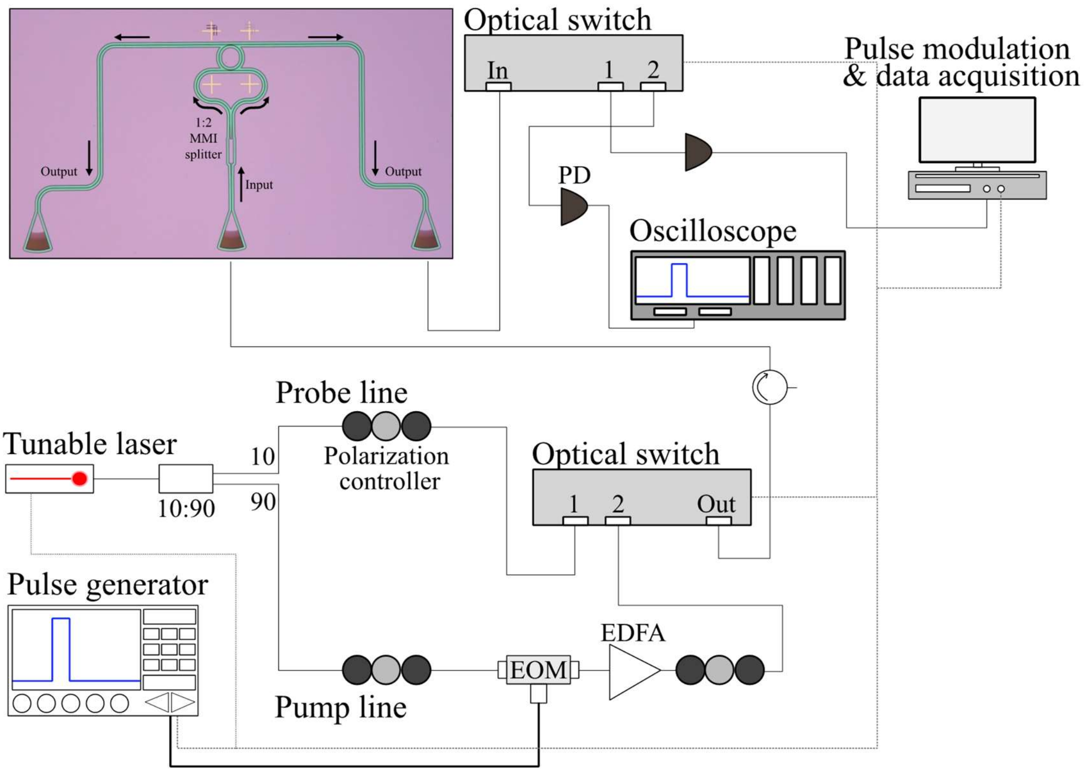

Supplementary Fig. 4. Fiber-optical pump/probe measurement setup to characterize PSR. Pump and probe lines are isolated by optical switches. Tunable laser is used to measure optical transmission spectra in a probe mode, and also to switch the GST by high-power pulses controlled by pulse generator. Light is coupled in and out of integrated photonic circuit using grating couplers.

Section S1. Experimental transmission spectra of a PSR system

Our mircroring resonators exhibit intrinsically high-quality factor of ~5700 (Supplementary Fig. 5), making the overall system susceptible to fabrication or index variations. Accordingly, one can observe the blueshifts transmission peaks in Figure 2F after hot-plate annealing, which is related with evaporation of solvent or residue causing slight reduction in effective refractive index.[1] Such low fabrication tolerance can be overcome by post-processing techniques,[2-4] especially lossless, high-precision trimming method using the volume shrinkage of polymer layer.[5] Picometer-precision control of polymer thickness can finely adjust the spectral resonance positions of multi-resonator photonic circuits. Alternatively, the slight mismatch of spatial position of standing-waves can be further improved by using additional phase shifters, which we will revisit (in Section S3).

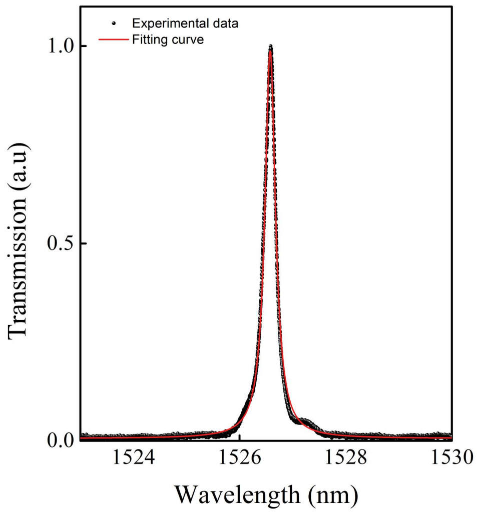

Supplementary Fig. 5 Transmission spectrum of a microring resonator with aGST nanoantennas and Lorentzian fitting curve demonstrating a quality factor of ~5700.

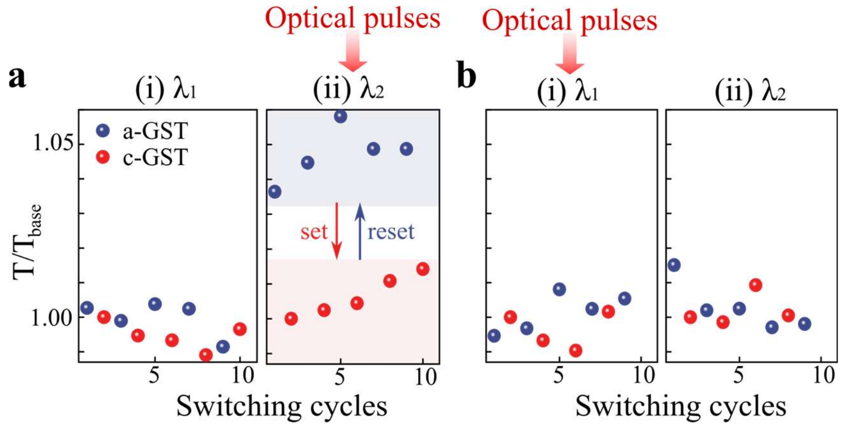

Supplementary Fig. 6 Wavelength-selective active switching of GST/Au nanoantennas with optical pulses at different wavelengths. λ_1 (1533.5 nm) is illuminated for **a** and λ_2 (1539.5 nm) is illuminated for **b**.

Section S2. Modulation contrast between wavelength-selective channels

Although we achieve the modulation contrast of ~40% and ~10% from hot-plate annealing (Figure 2F) and optical switching (Figure 3), respectively, this contrast can be further increased by optimizing the size and number of nanoantennas. We present the simulated transmission spectra of our PSR system with varying the size and number of nanoantennas (Supplementary Fig. 7). At first, a single nanoantenna with width of 100 nm and length of 250 nm exhibits ~53.2% and 14.8% when it gets fully or partially crystalline (50% crystalline) states, respectively (Supplementary Fig. 7A). Partially-crystalline state refers to the estimated scenario for optically switched state. This attenuation becomes more pronounced as three nanoantennas are placed on the nodes of on-resonance (λ_2) standing-waves (Supplementary Fig. 7B), while the non-trivial changes in off-resonance peak (λ_1) are observed due to slight spatial overlaps between the nanoantennas and off-resonance standing-waves. This undesirable absorption at off-resonance can be minimized by reducing the size of nanoantennas (Supplementary Fig. 7C-D). Overall, increasing the number of nanoantennas with smaller widths (Supplementary Fig. 7D) help increase the modulation contrast (57.0% for fully-crystalline, 17.2% for partially-crystalline states) without significantly degrading the crosstalk (7.1% for fully-crystalline, 1.2% for partially-crystalline states). Although we chose three nanoantennas as an example of demonstration, this can be further enhanced with fine-tuning their size, thickness or material choices. For instance, the use of phase-change material with larger extinction constant (eg, $Ge_4Sb_6Te_7$)[6] or low-loss tunable material (eg, Sb_2Se_3)[7,8] can achieve larger modulation contrast. In an ultimate case, the position of resonances can be shifted in a wavelength-selective manner so that much higher contrast can be attained with low crosstalk. Alternatively, electrical integration[9, 10] can enhance the modulation depth by directly measuring the photocurrent readout[10] or induce electrical indirect switching[9] at the expense of

optical loss from metallic electrodes.

Furthermore, the evanescent coupling between the guided standing-wave and GST nanoantennas can be enhanced to achieve larger absorption contrast. This can be accomplished by utilizing photon-plasmon coupling[6,11] with extra metal layer or partially-open slot waveguide.[12] While we have explored both approaches (see Methods) to increase the absorption of GST nanoantenna in Fig. 3 and Fig. 4, these can be further optimized by tailoring the dimension and shape of metallic layer or the geometries of slot waveguide.

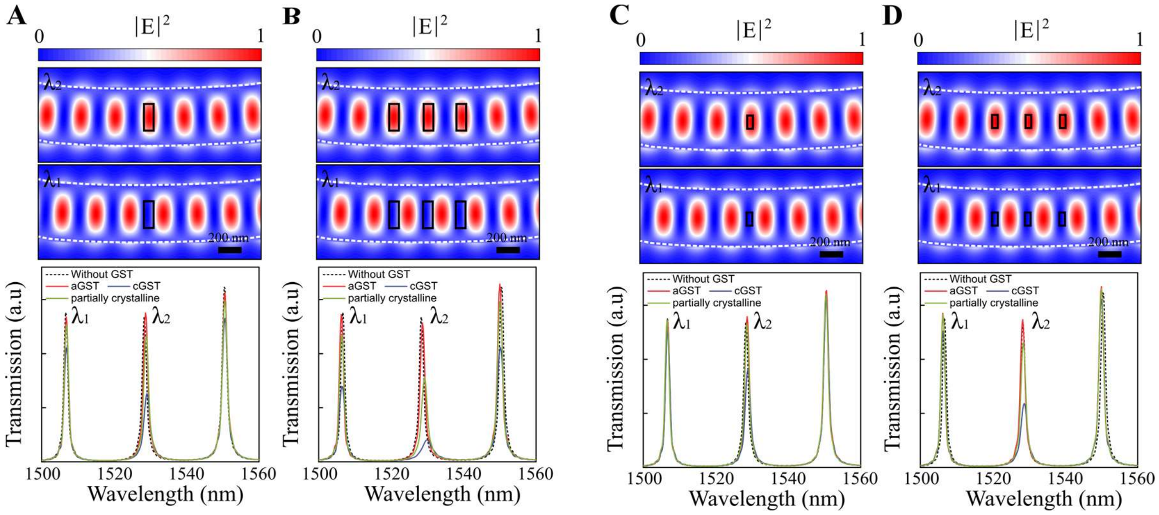

Supplementary Fig. 7 Spectral selectivity with varying size and number of nanoantennas. Enlarged field-distribution at on-resonance (λ_1 – *first row*) and off-resonance (λ_2 – *second row*) when the nanoantennas are positioned at the nodes of on-resonance electric-field. Transmission spectra (*third row*) of a bare ring resonator and the ones with nanoantennas are simulated, while the material states of nanoantenna are in amorphous, partially crystalline (50% crystalline) or fully crystalline phases. The nanoantennas with widths of 100 (65) nm and length of 250 (130) nm are demonstrated in **A-B** (**C-D**). Each device consists of 1 (*left column*) and 3 (*right column*) nanoantennas.

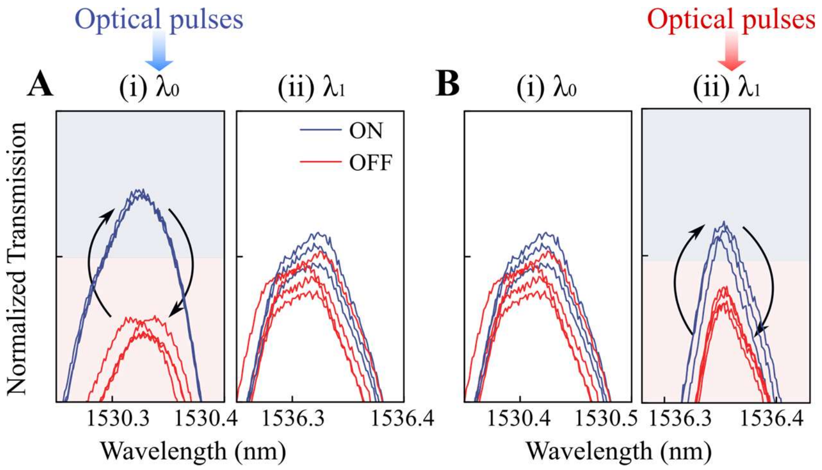

Supplementary Fig. 8. Enlarged transmission spectra of a microring resonator with PSR, demonstrating reversible switching cycles under **(a)** even- (λ_0, 1530. 5 nm) and **(b)** odd-mode (λ_1, 1536.4 nm) illumination with set (220 pJ for 10 ns + 1.8 nJ for 300 ns with repetition of 50 times) and reset (220 pJ for 10 ns) optical pulses.

Section S3. Control of optical phase onto a PSR system

Along with wavelength selective modulation, our PSR system possesses additional advantages of controlling spatio-spectral responses with using relative optical phase shifts between two input sources. By modulating the optical phase of one input arm by electro- or thermo-phase shifters (Supplementary Fig. 9), we can modify the interference conditions of two counter-propagating light, thereby tuning the spatial positions of standing-waves within a ring resonator. This spatial offset varies the overlap between standing-waves and nanoantennas as shown in Supplementary Fig. 10. One can observe that as the phase delay of one arm ($\Delta\Phi$) from 0° to 180°, the left nanoantenna becomes transparent to fully absorptive while the right nanoantenna shows the exactly opposite behavior. By continuously tuning the position of standing waves while the one of nanoantenna is fixed, the spatial overlap can be optimized. This feasible approach is expected to not only enhance the robustness of our system by optimizing any spatial mismatch between nanoantennas and standing-waves, but also add extra tunable functionalities in phase domain. In Supplementary Fig. 11, we experimentally demonstrate the above concept by finely sweeping the position of nanoantennas while those of standing waves are the same. Although some fluctuation exists due to low fabrication tolerances of microring resonators, one can see the same trend of transmission intensity variation as the nanoantenna gets shifted by $\lambda/2n_{eff}$ (i.e. 360°).

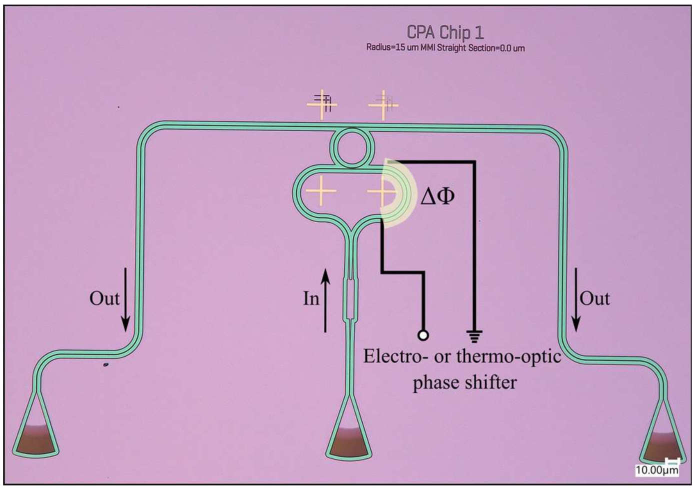

Supplementary Fig. 9. Optical microscope image of a PSR system with schematic illustration of electro- or thermo-optic phase shifter. Electronically programmable phase shifters can be integrated onto the one of input arms of a microring resonator to precisely tune the spatial position of standing-waves.

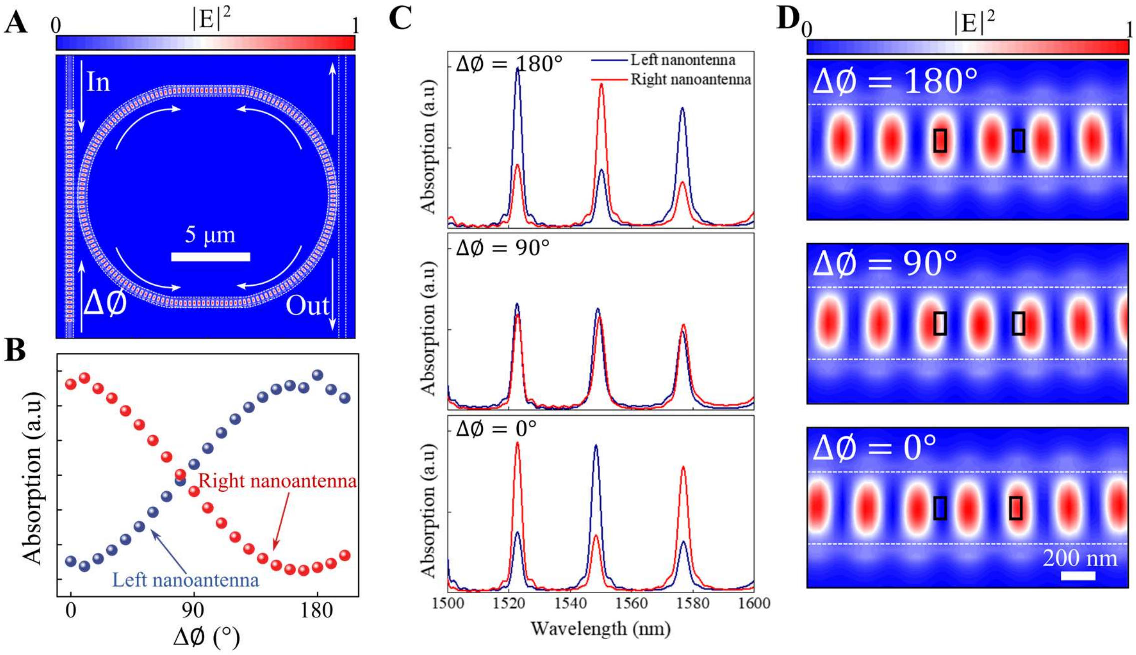

Supplementary Fig. 10. Spatio-spectral reconfiguration with varying the optical phases of input arms. (**A**) Electric-field distribution of a microring resonator when the counter-propagating waves generate standing waves (**B**) 1D absorption intensity profile of each nanoantenna at resonance (1524 nm) with varying the optical phase (ΔΦ) of one input arm. (**C**) Absorption spectra of each nanoantenna and (**D**) enlarged electric-field distribution with varying ΔΦ from 0° to 180°.

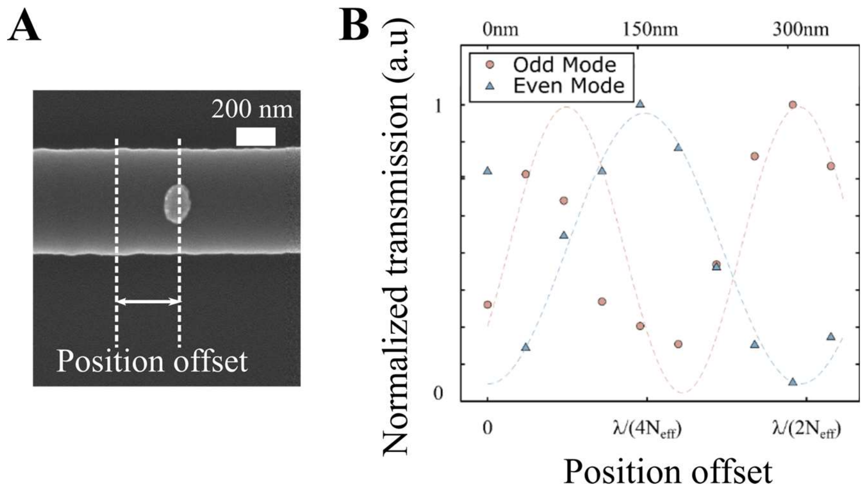

Supplementary Fig. 11. Transmission response of a PSR system with varying positions of nanoantennas. (**A**) Enlarged SEM scan of a microring resonator with nanoantenna deposited on. Transmission spectra of a PSR system are measured with increasing the position offset of the nanoantenna. (**B**) 1D transmission intensity profile of odd-mode (1550 nm) and even-mode (1555 nm) resonance with varying positions of the nanoantenna.

Section S4. Advantages of the PSR system in a large scale

Section S4.1. Spatio-spectral relation in a plain waveguide and microring resonator

The operational principle of the PSR relies on the spatio-spectral relation that the spatial position (P) of standing wave is directly proportional to its wavelength (λ) as shown below,

$$P = \lambda \cdot x / 2n_{eff} \quad (1)$$

where n_{eff} and x refer to the effective refractive index and position index along the waveguide (i.e. number of standing-wave cycles), respectively.

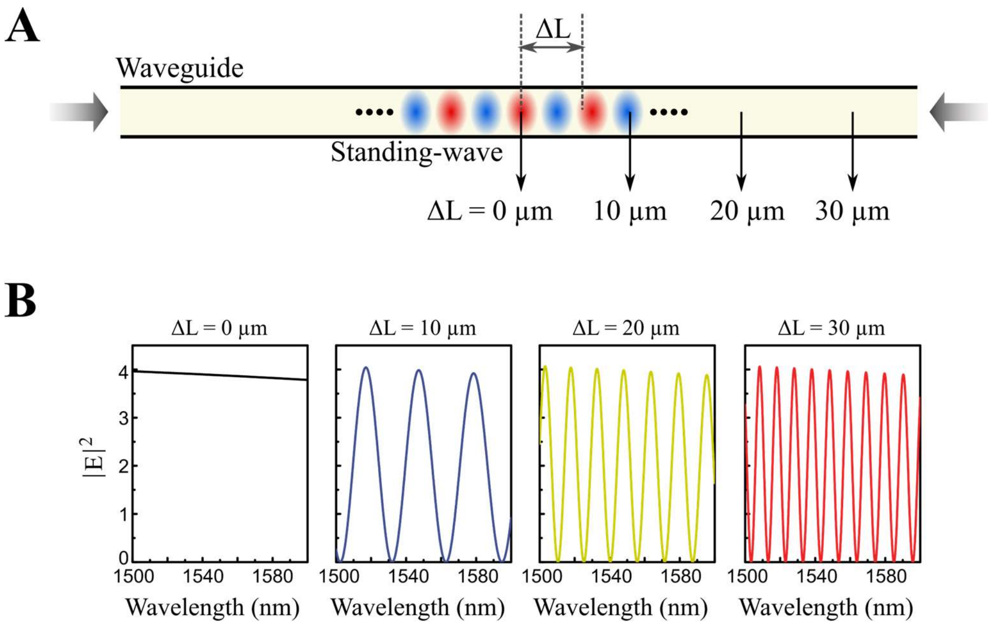

Supplementary Fig. 12. Wavelength-selective electric-field intensity in a plain waveguide. (**A**) Schematic diagram of a single Si waveguide when wavelength-selective standing waves are formed due to interference of counter-propagating light. ΔL denotes the distance away from the centre of two input sources. (**B**) Electric-field intensity as a function of wavelength at four different positions (ΔL) on a waveguide as denoted in (**A**).

If two counter-propagating waves are sent in a plain waveguide, there is negligible wavelength-selectivity in electric-field intensity at the centre of two light sources (Supplementary Fig. 12)

because standing waves interfere in the same manner regardless of their wavelengths. However, such selectivity becomes more pronounced as the offset distance (ΔL) increases due to the increased spatial mismatch between multi-wavelength standing waves. Therefore, the ideal modulation contrast in electric field intensities can be achieved by increasing either spatial (ΔL) or spectral (Δλ) separation.

For a microring resonator, the spatial positions of standing-waves are also determined by equation (1) for resonant wavelengths (λ_{res}= (*Ring Perimeter*·n_{eff})/*m*), where *m* is the resonance index of the microring. In this case, the standing waves of two adjacent resonant wavelengths (λ_m and $\lambda_{(m-1)}$) exhibit nearly out-of-phase electric-field intensity with each other, when the position index *x* is *m*/2. Consequently, the spatial positions (*P*) of odd- and even-mode standing waves (i.e. λ_m and $\lambda_{(m-1)}$) that exhibit the highest electric-field contrast with each other are *Ring Perimeter*/4 and *Ring Perimeter*/4 + $\lambda_{even}/4n_{eff}$, respectively. If the FSR of microring resonator is small enough, such mode-selective contrast can be achieved from every odd- and even-mode wavelength at the midpoint of symmetric microring resonator. This feature results in robust operation of two-wavelength modulation despite the spectral resonance variations in a microring resonator due to low fabrication tolerance.

While our experimental demonstration focuses on two-wavelength modulation using the analysis above, the relationship between spatial (ΔL) or spectral (Δλ) separation in Supplementary Fig. 12 raises the implication that the number of modulations can be extended beyond two by precisely selecting the spatio-spectral position that provides the minimum crosstalk selectivity between multiple standing waves in plain waveguides or microring resonators.

Section S4.2. Increasing the number of modulation channels in a single PSR system

Based on the above observations, we present feasible approaches to increase the number of independent modulation channels with our PSR devices. At first, we find the resonance positions (λ_{res}) for a microring resonator with its radius (R) of 15 μm within the given spectral range (1.5 – 1.6 μm). Then we calculate the periodicity (P_{res}) of resonant standing-waves and simulate their electric-field intensities ($E_{res}(x) = \cos(P_{res} \cdot x + \Delta\Phi)$) as a function of distance (x) along the half of ring perimeter ($R \cdot \pi$) of propagation axis, while $\Delta\Phi$ is fixed.

With using the simulated $E_{res}(x)$, we find the combination of three optimal resonances (λ_1, λ_2, λ_3) and the corresponding spatial positions (x_1, x_2, x_3), which results in the minimum crosstalk of electric-field intensity between the on-resonance and the other two off-resonance standing-waves (Supplementary Fig. 13-14). The crosstalk is defined by the ratio of electric-field intensity of standing wave at on-resonance to the sum of those at off-resonances. This calculation allows us to precisely locate the nanoantenna onto a standing-wave microring resonator. Three nanoantennas at each position show wavelength-selective absorption with the minimum crosstalk selectivity of <-15 dB. Therefore, wavelength-selective absorption (Supplementary Fig. 13C) enables independent switching up to three separate channels, and phase transition of nanoantennas can induce wavelength-selective change in readouts (Supplementary Fig. 13D). The crosstalk selectivity of < -13 dB is maintained with a fabrication resolution of ~ ±15 nm, which allows three independent wavelength modulations with 15 nm precision. The number of modulations can be further increased at the expense of size of microring, spectral range of modulation or size of nanoantennas. In Supplementary Fig. 13E, we vary each of these parameters, find the optimal spatio-spectral positions (x_n, λ_n) for lowest crosstalk with increasing the number of modulations from 2 to 4.

At first, as the size of a ring resonator increases, the light propagating distance increases with allowing for more overlap between on-resonance high-nodes and off-resonance low-nodes of standing-waves (Supplementary 13E, *left*). Also, increasing ring radii consequently decreases free-spectral range (FSR), resulting in more resonances for a given spectral range. This provides more combinations of various resonances, thereby reducing the crosstalk of the system. In some cases, especially for two wavelength modulation, smaller FSR contributes to smaller discrepancies in the period of standing-waves which can improve their selectivity. On the other hand, for larger numbers of modulation, the crosstalk can be further minimized by expanding the spectral range of modulation (normally determined by the types of optical couplers) because the number of wavelength selections accordingly increases with enlarged spatial offsets between standing-waves (Supplementary 13E, *middle*). However, as the size of nanoantenna increases, non-trivial absorption at off-resonances degrades the overall crosstalk (Supplementary 13E, *right*). This limitation can be further improved by expanding a variety of material choices from lossy chalcogenide to low-loss material. By varying these parameters, more than four modulations can be also achieved with low modal crosstalk, and such compact multiplexing functionality enables significant enhancement in optical storage density as the system gets upscaled (Section S4.3).

It is important to highlight that the low fabrication tolerance of microring resonators leads to variations in spectral resonance positions, posing a challenge in predicting the exact spatial positions of standing waves. This can be a limiting factor to precisely position the nanoantennas at the optimum location and achieve a robust multi-spectral modulator when the number of modulations exceeds two. This limitation, however, can be mitigated by employing fine-tuning methods, such as sub-nanometer-precision polymer trimming method[5] (Supplementary Section

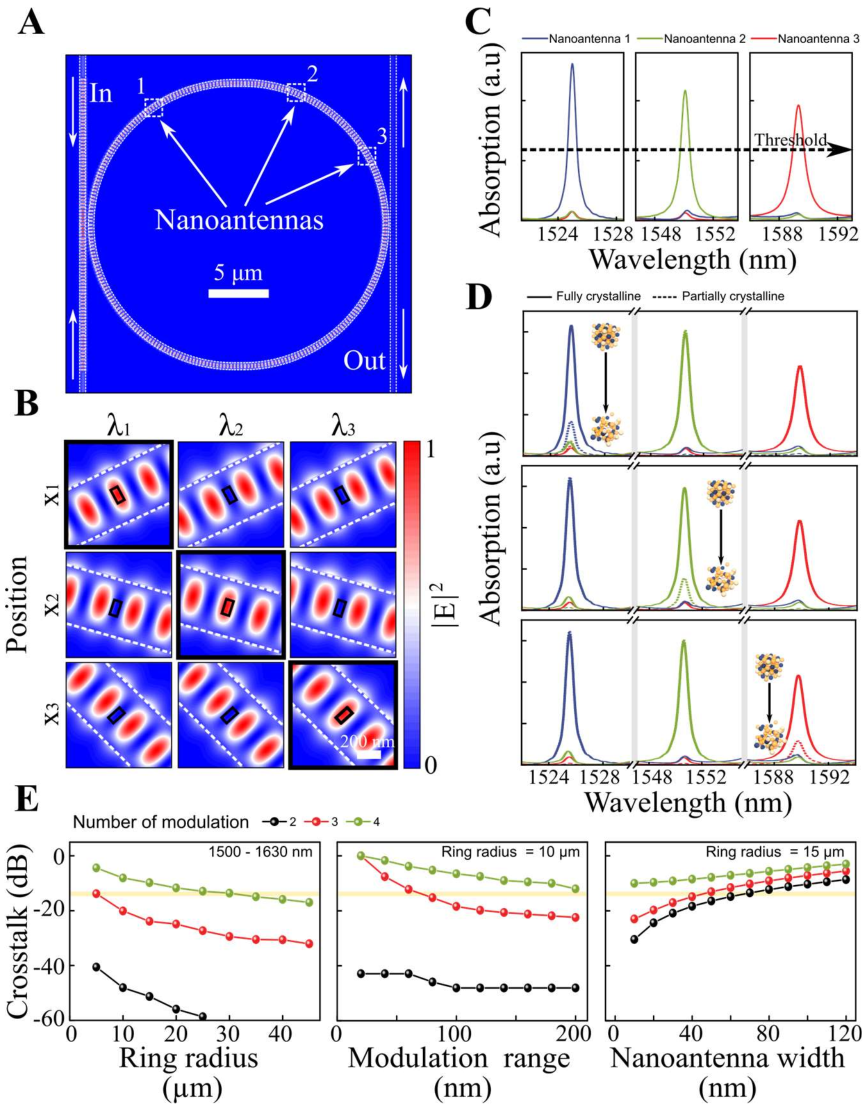

Supplementary Fig. 13. Multiple modulation in a single ring (**A-B**) Electric-field distribution of a microring resonator with counter-propagating waves from two input channels. Radius of the ring is 15 μm. Three nanoantennas are placed on three different positions (x_1, x_2, x_3) of the ring under illumination of three distinct wavelengths (λ_1, λ_2, λ_3). Enlarged field profile shows that each nanoantenna is spatially overlapped with the standing-waves of corresponding wavelengths (*outlined in bold*), while the others do not interact with the standing-waves. Note that additional three nanoantennas are positioned at the opposite side of a microring to balance the optical path length (**C-D**) Simulated absorption spectrum of each nanoantenna at three different wavelengths. While each nanoantenna *(top* – 1, *middle* – 2, *bottom* – 3) switches its state from fully crystalline to 50% crystalline with others remaining in fully crystalline, the absorption spectra selectively show the intensity changes on the nanoantenna of interest. (**E**) Calculated crosstalk as a function of (*left*) ring radius, (*middle*) modulation range and (*right*) widths of nanoantennas, when the number of modulation is increased from 2 to 4. Yellow lines refer to crosstalk of -15 dB as a reference.

S1) for optimizing spectral position and thermo-optical phase shifts (Supplementary Section S3) for adjusting spatial position of standing-waves.

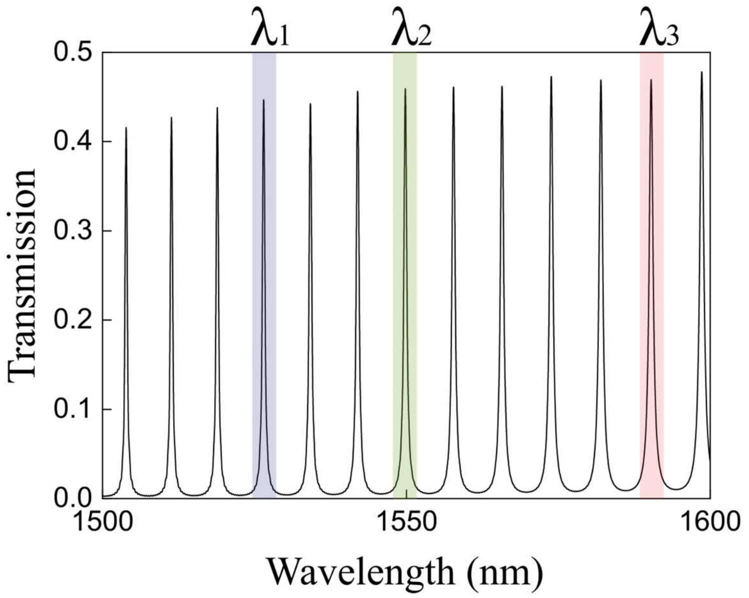

Supplementary Fig. 14. Transmission spectra of a microring resonator with the radius of 15 μm. Three resonant wavelengths (λ_1, λ_2, λ_3) correspond to the independent modulation channels in Supplementary Fig. 13.

Section S4.3. Advantages of a large-scale PSR system in multiplexing and modulation

Despite the tradeoff, one can see the feasibility of controlling three independent wavelengths with the crosstalk of -16~19 dB, using viable device parameters: the ring radius of 15 μm, the modulation spectral range 66 nm (1524 – 1590 nm) and the nanoantenna width of 65 nm. This selectivity is found to be comparable to other integrated (de)multiplexer such as angled multimode interferometer,[13] integrated ring demultiplexer,[14,15] or mode-division multiplexers,[16,17] while our system operates at a single photonic element. Therefore, our devices exhibit larger modulation density (number of modulation · modulation depth/device

footprint) or multiplexing density (number of modulation · crosstalk/device footprint) than other above-mentioned designs. Additionally, the non-volatile tunability of our active nanoantennas enables the full operation with zero static energy, achieving energy-efficient modulators compared to other electro- or thermo-optic switches. Programming energy (~220 pJ, Figure 4) can be further minimized by strongly confining electric-field within the volume of phase-change by plasmonic nanogap[18] or photonic-crystal microcavities.[19] Modulation bandwidth of our system is limited by the crystallization time of phase-change material, but most programming can be done using 10 – 25 ns pulses (Figure 4) with achieving 50 – 100 MHz. The thermo-optic heat diffusion time from the GST and silicon waveguide can be the limiting factor for fast operation,[20] however the delay time of submicrons-sized GST nanoantenna is expected to be minimal considering that the 4 μm-long GST layer on a silicon waveguide exhibits few tens of nanometer dead time.[21] This can be further improved to GHz-THz regime by using pico- or femtosecond optical pulses[22,23] or exploiting the phase-change nanoantennas as wavelength-multiplexed in-memory units[24] which enhance modulation bandwidths by the number of modulation channels with electro-optical modulators.

These advantages of PSR system can become more beneficial as it gets upscaled, for instance, by assembling multiple microring resonators in series. As shown in Supplementary Fig. 15, conventional photonic integrated circuits (PICs) require three ring resonators with different ring radii for three independent modulations. In this case, based on a permutation scheme, the maximum number of device states are defined as 2^N and M^N for binary and multilevel modulation, respectively, whereas N and M refer to the number of rings and distinct multi-levels, respectively. On the other hand, for standing-wave PICs (Supplementary Fig. 15, *bottom*), two modulation per ring can enhance the optical storage limit to 2^{2N} and M^{2N} for

binary and multi-level modulation, respectively. In this case, the enhancement factor is found to be M^2, while this can be even further increased to M^n, by combining the concept of multiple modulations per microring (n) as described in Section 4.2. Overall, the confined multiplexing functionality in a large-scale PSR system is expected to dramatically enhance the optical storage capacity, as the number of multilevels (M) or modulations (n) per microring increases.

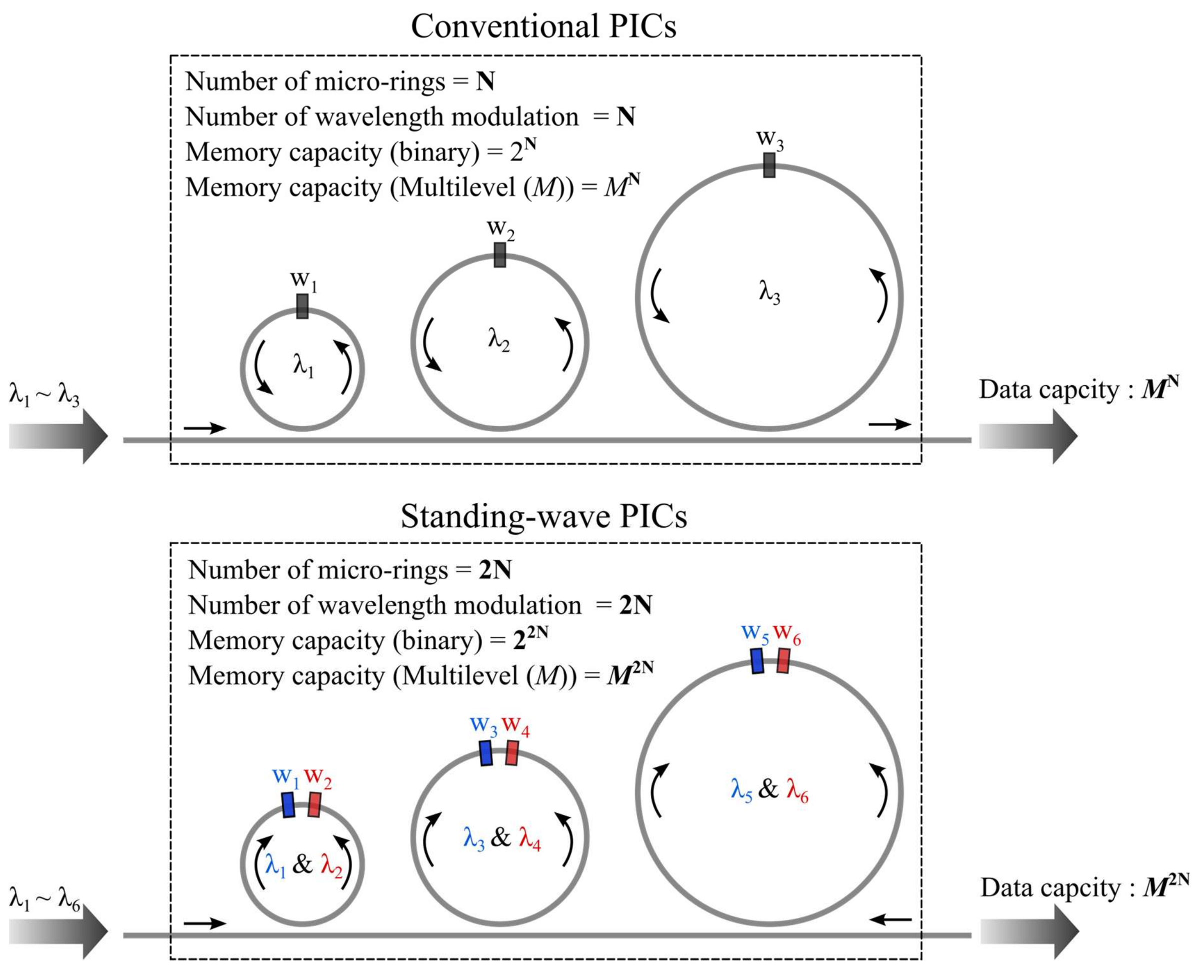

Supplementary Fig. 15. Schematic demonstration of optical storage density based on permutation scheme for (*top*) conventional and (*bottom*) standing-wave photonic integrated circuits. N and M refers to the number of ring resonators and multi-level states per ring, respectively. Three ring resonators with different radii were demonstrated as an example.

References

1 Song, S., Dua, J. & Arnold, C. B. Influence of annealing conditions on the optical and structural properties of spin-coated As2S3 chalcogenide glass thin films. *Optics Express* **18**, 5472-5480 (2010).

2 Jayatilleka, H. *et al.* Post-Fabrication Trimming of Silicon Photonic Ring Resonators at Wafer-Scale. *Journal of Lightwave Technology* **39**, 5083-5088 (2021).

3 Wu, Y., Shang, H., Zheng, X. & Chu, T. Post-Processing Trimming of Silicon Photonic Devices Using Femtosecond Laser. *Nanomaterials* **13** (2023).

4 Biryukova, V., Sharp, G. J., Klitis, C. & Sorel, M. Trimming of silicon-on-insulator ring-resonators via localized laser annealing. *Optics Express* **28**, 11156-11164 (2020).

5 Farmakidis, N. *et al.* Scalable High-Precision Trimming of Photonic Resonances by Polymer Exposure to Energetic Beams. *Nano Letters* **23**, 4800-4806 (2023).

6 Kusne, A. G. *et al.* On-the-fly closed-loop materials discovery via Bayesian active learning. *Nature Communications* **11**, 5966 (2020).

7 Ríos, C. *et al.* Ultra-compact nonvolatile phase shifter based on electrically reprogrammable transparent phase change materials. *PhotoniX* **3**, 26 (2022).

8 Xu, P., Zheng, J., Doylend, J. K. & Majumdar, A. Low-Loss and Broadband Nonvolatile Phase-Change Directional Coupler Switches. *ACS Photonics* **6**, 553-557 (2019).

9 Farmakidis, N. *et al.* Electronically Reconfigurable Photonic Switches Incorporating Plasmonic Structures and Phase Change Materials. *Advanced Science* **9**, 2200383 (2022).

10 Schuler, S. *et al.* High-responsivity graphene photodetectors integrated on silicon microring resonators. *Nature Communications* **12**, 3733 (2021).

11 Chen, B. *et al.* Hybrid Photon–Plasmon Coupling and Ultrafast Control of Nanoantennas on a Silicon Photonic Chip. *Nano Letters* **18**, 610-617 (2018).

12 Ritter, R. *et al.* Coupling Thermal Atomic Vapor to Slot Waveguides. *Physical Review X* **8**, 021032 (2018).

13 Chen, J., Liu, P. & Shi, Y. An on-chip silicon compact triplexer based on cascaded tilted multimode interference couplers. *Optics Communications* **410**, 483-487 (2018).

14 Jayatilleka, H. *et al.* Crosstalk in SOI Microring Resonator-Based Filters. *Journal of Lightwave Technology* **34**, 2886-2896 (2016).

15 Xu, H., Liu, C., Dai, D. & Shi, Y. Direct-access mode-division multiplexing switch for scalable on-chip multi-mode networks. *Nanophotonics* **10**, 4551-4566 (2021).

16 Luo, L.-W. *et al.* WDM-compatible mode-division multiplexing on a silicon chip. *Nature Communications* **5**, 3069 (2014).

17 Jia, H. *et al.* Microring modulator matrix integrated with mode multiplexer and de-multiplexer for on-chip optical interconnect. *Optics Express* **25**, 422-430 (2017).

18 Farmakidis, N. *et al.* Plasmonic nanogap enhanced phase-change devices with dual electrical-optical functionality. *Science Advances* **5**, eaaw2687.

19 Foresi, J. S. *et al.* Photonic-bandgap microcavities in optical waveguides. *Nature* **390**, 143-145 (1997).

20 Stegmaier, M., Ríos, C., Bhaskaran, H. & Pernice, W. H. P. Thermo-optical Effect in Phase-Change Nanophotonics. *ACS Photonics* **3**, 828-835 (2016).

21 Li, X. *et al.* Experimental investigation of silicon and silicon nitride platforms for phase-change photonic in-memory computing. *Optica* **7**, 218-225 (2020).

22 Feldmann, J. *et al.* Calculating with light using a chip-scale all-optical abacus. *Nature Communications* **8**, 1256 (2017).

23 Loke, D. *et al.* Breaking the Speed Limits of Phase-Change Memory. *Science* **336**, 1566-1569 (2012).

24 Feldmann, J. *et al.* Parallel convolutional processing using an integrated photonic tensor core. *Nature* **589**, 52-58 (2021).
